# Improving the Field’s Understanding of Suicide Protective Factors and Translational Suicide Prevention Initiatives

**DOI:** 10.3390/ijerph18031027

**Published:** 2021-01-25

**Authors:** Robert J. Cramer, Raymond Tucker

**Affiliations:** 1 Department of Public Health Sciences, UNC Charlotte, Charlotte, NC 28233, USA; 2 Department of Psychology, Louisiana State University, Baton Rouge, LA 70803, USA; rtucker1@lsu.edu

World Health Organization data show that approximately 800,000 persons die by suicide each year [1]. Moreover, suicide trends remain stable or are increasing across many nations. In light of the global concern posed by suicide, decades of research have been devoted to identifying factors that place people at risk of suicidal thoughts and behaviors (STBs). Despite these efforts, a recent meta-analysis demonstrated that most risk factors do little better than chance in predicting future STBs [2]. Suicide resilience factors were not better predictors of suicide either; however, they have been dramatically understudied according to this synthesis of the literature. Thus, this Special Issue includes articles that attempt to increase the field’s understanding of suicide resilience. Clement and colleagues [3] investigate the interrelationships between hope, optimism, hopelessness, and grit, as well as their collective relationship to suicidal ideation. In a cross-sectional sample of undergraduate college students, the authors used exploratory factor analytic techniques to determine how these risk and resilience factors relate at both the subscale and item level. The authors demonstrated a single-factor solution for these measures when subscale scores were entered; however, when all items across the scales were factor analyzed together, five separate subscales emerged that did not represent the original five scales. These newly defined subscales had differential relationships with suicidal ideation, demonstrating that a potential reconceptualization of positive future thinking styles may be needed to better understand suicide resilience.

The presence of positive future thinking such as hope is a clear correlate of indicators of mental well-being in a variety of samples [4]. Russel, Rasmussen, and Hunter [5] extend research on mental well-being and risk of STBs in a longitudinal sample of adolescents assessed at baseline and 6-month follow-up. Their investigation demonstrates that mental well-being appears protective against eventual thoughts of self-harm and self-harm behaviors through a reduction in feelings of defeat and entrapment. This investigation through the lens of the Integrated Motivational Volitional model of suicide (IMV) [6] provides a useful framework of understanding how upstream suicide prevention efforts that foster broader mental well-being may impact risk of self-harm and suicide.

Although a broad framework such as IMV may inform suicide prevention efforts, unique resilience factors exist in underrepresented populations [7]. The integration of these group-specific factors with information derived from majority populations may increase the relevance of suicide resilience frameworks in underrepresented groups [8]. Cramer and colleagues integrate identity characteristics into the central tenets of the preferences in information processing (PIP) model of suicide risk in a sample of adults who self-identify as members of the alternative sexuality community [9]. Higher education and monogamous relationship status were suicide protective factors. More importantly, a high need for affect approach, or a willingness to engage emotions, buffered the negative association between depression and suicide.

The three investigations noted above attempt to inform the suicide resilience research literature. Despite a disparity in work that investigates suicide resilience compared to suicide risk [10], research demonstrates some progress in the creation of several evidence-based, suicide-specific interventions such as Dialectical Behaviour Therapy (DBT) [11], Brief Cognitive Behavioral Therapy (BCBT) [12], and the Collaborative Assessment and Management of Suicide (CAMS) [13]. The promising evidence of these interventions in reducing STBs is met with a paucity of research regarding the actual use of suicide-specific care in the community. Moscardini and colleagues [14] investigate the extent to which behavioral health providers routinely use suicide safety planning [15] in their everyday practice. The investigation reveals a high level of comfort and the regular use of the intervention, but an overarching desire for continued training in suicide safety planning. Similarly, the investigation shows that the frequency of utilization of the intervention varies across providers and is related to providers’ personal history of experiencing STBs but not professional exposure to patient suicide. This research indicates that, although suicide safety planning may be a vitally important skill in any behavioral health provider’s preverbal toolbox, the successful implementation of this intervention is not a given and continued education in the intervention may be needed for successful regular utilization.

Finally, suicide-specific care such as suicide safety planning is not always needed in treating patients presenting for mental health concerns. Thus, although suicide prediction appears to be a difficult pursuit [16], suicide risk identification is a necessary step to determine the level of suicide-specific care that may be warranted. Cohen and colleagues [17] conducted a feasibility investigation of whether machine learning techniques with natural language processing of adolescent discussions with their therapists may improve suicide risk assessment practices. The investigation demonstrated that this practice focused on augmenting standardized clinical risk assessment efforts may provide important information for assessing risk of STBs and thus the cueing of suicide-specific care practices.

In summary, this Special Issue of the *International Journal of Environmental Research and Public Health* details new investigations into factors that relate to suicide resilience in samples of adolescents and adults. This research indicates a need for the further refinement of our knowledge of how positive thinking styles relate to each other and thus suicide resilience, the need for upstream suicide prevention efforts that target not only risk of STBs but broader mental well-being, and the utility of incorporating identity considerations into theoretical frameworks of suicide risk and resilience. This research could inform the refinement and creation of suicide-specific interventions. This work, however, should be aware of important implementation considerations identified by providers using suicide safety planning. It is possible for behavioral healthcare providers to feel confident in their utilization of these interventions but demonstrate varying indicators of using these practices with fidelity. Finally, suicide risk assessment is needed to identify the level of suicide-specific care a patient may need. The use of natural language processing and machine learning techniques may enhance the identification of the level of care needed.

## References

[1] Värnik P. (2012). Suicide in the world. Int. J. Environ. Res. Public Health.

[2] Franklin J.C., Ribeiro J.D., Fox K.R., Bentley K.H., Kleiman E.M., Huang X., Musacchio K.M., Jaroszewski A.C., Chang B.P., Nock M.K. (2017). Risk factors for suicidal thoughts and behaviors: A meta-analysis of 50 years of research. Psychol. Bull..

[3] Clement D.N., Wingate L.R., Cole A.B., O’Keefe V.M., Hollingsworth D.W., Davidson C.L., Hirsch J.K. (2020). The common factors of grit, hope, and optimism differentially influence suicide resilience. Int. J. Environ. Res. Public Health.

[4] Lee J.Y., Gallagher M.W. (2018). Hope and well-being. The Oxford Handbook of Hope.

[5] Russell K., Rasmussen S., Hunter S.C. (2020). Does mental well-being protect against self-harm thoughts and behaviors during adolescence? A six-month prospective investigation. Int. J. Environ. Res. Public Health.

[6] O’Connor R.C., Kirtley O.J. (2018). The integrated motivational–volitional model of suicidal behaviour. Phil. Trans. R. Soc. B Biol. Sci..

[7] Chu J.P., Goldblum P., Floyd R., Bongar B. (2010). The cultural theory and model of suicide. Appl. Prev. Psychol..

[8] Tucker R.P. (2019). Suicide in transgender veterans: Prevalence, prevention, and implications of current policy. Pers. Psychol. Sci..

[9] Cramer R.J., Langhinrichsen-Rohling J., Kaniuka A.R., Wilsey C.N., Mennicke A., Wright S., Montanaro E., Bowling J., Heron K.E. (2020). Preferences in information processing, marginalized identity, and non-monogamy: Understanding factors in suicide-related behavior among members of the alternative sexuality community. Int. J. Environ. Res. Public Health.

[10] O’Connor R.C., Nock M.K. (2014). The psychology of suicidal behaviour. Lancet Psychiatry.

[11] Linehan M.M., Korslund K.E., Harned M.S., Gallop R.J., Lungu A., Neacsiu A.D., McDavid J., Comtois K.A., Murray-Gregory A.M. (2015). Dialectical behavior therapy for high suicide risk in individuals with borderline personality disorder: A randomized clinical trial and component analysis. JAMA Psychiatry.

[12] Rudd M.D., Bryan C.J., Wertenberger E.G., Peterson A.L., Young-McCaughan S., Mintz J., Williams S.R., Arne K.A., Breitbach J., Delano K. (2015). Brief cognitive-behavioral therapy effects on post-treatment suicide attempts in a military sample: Results of a randomized clinical trial with 2-year follow-up. Am. J. Psychiatry.

[13] Andreasson K., Krogh J., Wenneberg C., Jessen H.K., Krakauer K., Gluud C., Thomsen R.R., Randers L., Nordentoft M. (2016). Effectiveness of dialectical behavior therapy versus collaborative assessment and management of suicidality treatment for reduction of self-harm in adults with borderline personality traits and disorder—A randomized observer-blinded clinical trial. Depress. Anxiety.

[14] Moscardini E.H., Hill R.M., Dodd C.G., Do C., Kaplow J.B., Tucker R.P. (2020). Suicide safety planning: Clinician training, comfort, and safety plan utilization. Int. J. Environ. Res. Public Health.

[15] Stanley B., Brown G.K. (2012). Safety planning intervention: A brief intervention to mitigate suicide risk. Cogn. Behav. Prac..

[16] McHugh C.M., Corderoy A., Ryan C.J., Hickie I.B., Large M.M. (2019). Association between suicidal ideation and suicide: Meta-analyses of odds ratios, sensitivity, specificity and positive predictive value. BJPsych Open.

[17] Cohen J., Wright-Berryman J., Rohlfs L., Wright D., Campbell M., Gingrich D., Santel D., Pestian J. (2020). A feasibility study using a machine learning suicide risk prediction model based on open-ended interview language in adolescent therapy sessions. Int. J. Environ. Res. Public Health.

